# Supplementary material for: Patterns of Intron Gain and Loss in Fungi
Source: PLoS Biol. 2004 Nov 30;2(12):e422. doi: 10.1371/journal.pbio.0020422 (PMC532390; doi:10.1371/journal.pbio.0020422)
Supplement: Table S1 — Also available at http://genes.mit.edu/NielsenEtAl/. (4.3 MB ZIP). [file pbio.0020422.st001.zip › NielsenEtAl/html/538.html]

AN2977.1.NCU07465.1.MG09906.1.FG01230.1


```
 CLUSTAL W (1.82) Multiple Sequence Alignments - Introns Inserted


Sequence 1: NCU07465.1	382 aa
Sequence 2: FG01230.1	378 aa
Sequence 3: MG09906.1	321 aa
Sequence 4: AN2977.1	378 aa
Alignment Length: 387 aa
Number Identitical Residues: 199 aa
Alignment Score (without introns) 9581


MG09906.1 	MANVSSTRGPLP------------------------------------------------
NCU07465.1	MPSVYPGLDILQNTFGSSYQPKSNGATPLQ--ARTDLYPAYS-IVDDAKSKAKKLSAEAA
FG01230.1 	MSPLHPSKDALQGMFYSSLSATN--HTPYQ--ARAELFPIHSSVVEDAKGKAKKLSAEAT
AN2977.1  	---MFPPQDVMSGAFSAQRQWSQNQQTLGTGPTRREPFPAWS-VVEDTKKKANAISKEAA
          	   : .  . : .   :. . ...  :   ..:  .  .  :   ..:. .:.  : .::

MG09906.1 	-----------GPNGKIELYSGQYYASCAIGGLLAC0GLTHAAVTPLDLVKCRRQVDAKL
NCU07465.1	HEFDVASQKAQAKTGHIELYSSQYYAACTFGGLMAC0GVTHTAVTPLDLVKTRRQIDSKL
FG01230.1 	AEFEKASAKAQAKTGKIELYSGRYYTACTFGGLMAC0GLTHAAVTPLDLVKTRRQVDSKL
AN2977.1  	REFEIASQKAQSKTGKIEPWSAKYYAACTFGGLLAC0GLTHTAVTPLDLIKCRRQVDSAL
          	 . . :: .:.. .*:** :*.:**::*::***:** *:**:*******:* ***:*: *

MG09906.1 	YKSNLQGWSVIYRGEGIRGIFTGWSPTLFGYS~AQGAFKYGFYEFFKKKYSDVVGPEYAH
NCU07465.1	YKGNFQAWGHILRHEGVRGIFTGWSPTFFGYS~AQGAFKYGWYEFFKKKYSDLAGPENAH
FG01230.1 	YTSNFQAWGKIYRGEGIRGIFTGWSPTLFGYS~AQGAFKYGWYEYFKKTYSDMAGPEAAT
AN2977.1  	YKSNMDAFRKIRAAEGLRGVFTGWSPTFFGYS0AQGAFKYGGYEYFKKFYSDLVGVENAA
          	*..*::.:  *   **:**:*******:**** ******** **:*** ***:.* * * 

MG09906.1 	QYKTGVYLAASASAELIADAALCPFEAVKVRMQTTIPAQYKGTFDGISQIMAKEGASG~-
NCU07465.1	KYKTALYLSASASAEFLADIALCPFEAIKVRMQGTIPSQYKGTFDGFSQITAKEGWGG~-
FG01230.1 	KYKTGLYLAASASAEFLADLALCPFEAVKVRMQGSIPNPYTGTVQGIGAITGKEGVAG~-
AN2977.1  	RYKTPLYLAASASAELIADVALCPFEAVKVRTQTTIPPEFRSTFGGISAVVAKEGTAG2A
          	:*** :**:******::** *******:*** * :**  : .*. *:. : .*** .* :

MG09906.1 	-LYKGIYPLWGRQIPYTMMKFASFETIVEMIYDRLPRAKDEYSKLAQTGVSFTGGYLAGI
NCU07465.1	-LYKGLYPLWGRQIPYTMMKFASFETIVEMIYDRLPGGKSDYGKAAQTGVSFVAGYAAGI
FG01230.1 	-LYKGLYPLWGRQIPYTMMKFASFETIVEMIYDRLPGQKSDYSKAAQTGVSFTGGYLAGI
AN2977.1  	RLYKGLYPLWGRQIPYTMMKFASFENIVEAIYHYLPGQKSDYNKGAQTAVAFTGGYLAGI
          	 ****:*******************.*** **. **  *.:*.* ***.*:*..** ***

MG09906.1 	LCAAVSHPADVMVSKLNVYREPGEAVGAVLSRVYGDIGFRGLWNGLPVRIVMVGTL~TGL
NCU07465.1	LCAIVSHPADVMVSKLNAYRKPGEGMGAVTSRIYKDIGFKGLWNGLPVRIVMIGTL~TGL
FG01230.1 	LCAIVSHPADVMVSKLNSNRQPGEAFGSAMGRIYKDIGFGGLWNGLPVRIVMIGTL~TGL
AN2977.1  	LCAIVSHPADVMVSKLNANRLPGEAFGAATGRIYKEIGFMGLWNGLPVRIIMIGTL0TGL
          	*** *************  * ***..*:. .*:* :*** **********:*:*** ***

MG09906.1 	QWMI~Y0DYFKIFMGFPTTGGSAKPKNE-----
NCU07465.1	QWMI~Y~DYFKVFMGLPTTGGAAPPAEKPAQHS
FG01230.1 	QWMI~Y0DYFKIFMGLPTTGGGPPPAKKQE---
AN2977.1  	QWMI2Y~DSFKIFMGLPTTGGGPAKEQK-----
          	**** * * **:***:*****..   ::
```
